# Image-Guided Adaptive Brachytherapy for Uterine Cancer: A Comprehensive Review

**DOI:** 10.3390/cancers18040693

**Published:** 2026-02-20

**Authors:** Yi-Ching Chen, Chi-Yuan Yeh

**Affiliations:** 1 Department of Medical Research, Tungs Taichung MetroHarbor Hospital, Wuchi District, Taichung 435403, Taiwan; cf0871@gmail.com; 2 Department of Radiation Oncology, Tungs Taichung MetroHarbor Hospital, Wuchi District, Taichung 435403, Taiwan

**Keywords:** brachytherapy, image-guided brachytherapy, radiotherapy, pelvic neoplasm

## Abstract

**Background/Objectives**: Image-guided adaptive brachytherapy (IGABT) has transformed the standard of care for locally advanced cervical cancer (LACC), enabling volumetric target definition and dose–volume histogram (DVH)-based planning to improve pelvic tumor control while limiting severe late toxicity. **Methods**: A comprehensive literature search of PubMed/MEDLINE and Embase was done for articles published up to August 2024, using combinations of the following keywords and Medical Subject Heading (MeSH) terms: “cervical cancer”, “endometrial cancer”, “vaginal cancer”, “uterine neoplasms”, “brachytherapy”, “high-dose-rate”, “image-guided”, “MRI-guided”, “3D brachytherapy”, “IGABT”, “interstitial”, “locoregional control”, “toxicity”, “quality of life”, and “patient-reported outcomes”. **Results**: We summarized the contemporary evidence on IGABT for cervical, endometrial, and primary or recurrent vaginal cancers, focusing on local control, survival, late morbidity, and patient-reported outcomes. We described the key target volume concepts (gross tumor volume, high- and intermediate-risk clinical target volumes), and the role of MRI-, CT-, and ultrasound-based planning with intracavitary, intracavitary–interstitial, and interstitial applicators. **Conclusions**: Image-guided adaptive brachytherapy has redefined the standard of care for the management of locally advanced cervical cancer. Through the integration of volumetric target concepts, DVH-based dose reporting, and advanced imaging, IGABT has enabled consistent dose escalation to the residual tumor while accounting for organ-at-risk constraints, resulting in high local control rates and reduced severe morbidity compared with historical 2D brachytherapy.

## Simple Summary

Brachytherapy is an essential part of curative treatment for cervical, endometrial, and vaginal malignancies. Over the past two decades, image-guided brachytherapy (IGABT) has transformed brachytherapy by allowing the dose to be tailored more precisely to the tumor in 3D while better protecting the surrounding organs. This has translated into improved local control and fewer severe long-term side effects in patients with locally advanced cervical cancer; similar results were seen in IGABT for endometrial and vaginal cancers. In this review, we summarize current evidence for IGABT across uterine cancers, discuss typical dose and volume concepts, and highlight patient-reported outcomes and emerging technologies such as advanced imaging and artificial intelligence. We also outline the practical challenges and opportunities for wider implementation of IGABT in diverse clinical settings.

## 1. Introduction

Cervical cancer is a leading malignancy worldwide, and concurrent chemoradiotherapy (CCRT) followed by added brachytherapy remains the standard curative approach for LACC [1,2]. Brachytherapy is an irreplaceable component of curative treatment, and is essential for delivering high doses of radiation to the primary tumor while exploiting a steep dose gradient to protect surrounding healthy tissues [3,4,5,6].

For decades, BT relied on the Manchester system and 2D planning based on orthogonal radiographs, using the Point A dose prescription and International Commission on Radiation Units and Measurements (ICRU) reference points [4,6,7,8,9,10]. This method proved inadequate, as it failed to account for patient-specific anatomical variability, tumor shrinkage during treatment, and the precise topographic changes between the tumor and adjacent critical structures, referred to as organs at risk (OARs) [3,4,5,6,10].

The introduction of IGABT marks a profound paradigm shift. Beginning in the early 2000s, the GYN GEC-ESTRO Working Group established foundational guidelines, ushering in an era of 3D image-based treatment planning [7,11,12,13,14,15]. IGABT enables adaptive management, allowing clinicians to adjust dose delivery based on the complex 3D topography of the residual tumor and OARs at the time of each fraction [5,8]. The goal of IGABT is to maximize the therapeutic ratio by achieving adequate dose coverage of the target volume (dose escalation) while maintaining OAR doses within established limits (dose de-escalation).

Against this background, image-guided brachytherapy (IGABT) is a central component of modern definitive and adjuvant radiotherapy for uterine malignancies [6,16,17,18]. However, most published reviews focus on locally advanced cervical cancer alone, and rarely integrate outcome data, patient-reported endpoints, and implementation issues across the broader spectrum of uterine cancers [19,20]. In this narrative review, we summarize contemporary evidence on IGABT for cervical, endometrial, and primary or recurrent vaginal cancers, with a particular focus on local control, survival, late toxicity, and quality of life [16,18]. We further highlight the technical principles of target and organ-at-risk delineation, dose–volume constraints, and fractionation, and discuss emerging developments in functional imaging, artificial intelligence, and radiomics [21,22]. Finally, we address the practical considerations for implementing IGABT across different resource settings and outline current evidence gaps and future research priorities [1,5,6,23].

## 2. Materials and Methods

### 2.1. Literature Search Strategy

We performed a narrative literature review of clinical and technical studies on image-guided brachytherapy for uterine malignancies, including cervical, endometrial, and primary or recurrent vaginal cancers. We conducted a comprehensive literature search of PubMed/MEDLINE and Embase for articles published up to August 2024, using combinations of the following keywords and Medical Subject Heading (MeSH) terms: “cervical cancer”, “endometrial cancer”, “vaginal cancer”, “uterine neoplasms”, “brachytherapy”, “high-dose-rate”, “image-guided”, “MRI-guided”, “3D brachytherapy”, “IGABT”, “interstitial”, “locoregional control”, “toxicity”, “quality of life”, and “patient-reported outcomes”. Reference lists of relevant reviews and key original articles were screened to identify additional studies. Clinical trial registries were consulted to capture ongoing or recently completed prospective studies when appropriate.

### 2.2. Study Selection and Data Extraction

We included original articles reporting clinical outcomes, toxicity, or patient-reported outcomes of IGABT in adult patients with cervical, endometrial, or vaginal cancers, treated with definitive or adjuvant intent. Both prospective and retrospective cohort studies, as well as relevant technical or dosimetric reports with clinical correlations, were considered. Case reports, small case series with fewer than 10 patients, pediatric studies, non-uterine gynecologic malignancies, articles not using image-guided brachytherapy techniques, and non-English publications were excluded. From each eligible study, we extracted information on patient and tumor characteristics, imaging and applicator techniques, dose and fractionation schedules, target and organ-at-risk dose–volume parameters, oncologic outcomes, late toxicity, and quality-of-life or patient-reported outcome measures when available.

## 3. Results

Image-guided adaptive brachytherapy is built on a common set of geometric and dosimetric principles that can be applied across cervical [11,12,13,14,16], endometrial [17,24,25,26], and vaginal cancers [27,28,29]. These principles link three key components:

1. Target volume concepts that capture residual macroscopic and microscopic disease;
2. Dose metrics that describe the dose–response relationship for tumor control and organs at risk (OARs);
3. Imaging and applicator selection that enable individualized, anatomy-adapted treatment delivery.

Although most of the evidence derives from locally advanced cervical cancer, the same framework can be adapted to postoperative endometrial cancer and primary or recurrent vaginal disease, with attention given to site-specific anatomy and patterns of spread [5,12,30,31].

### 3.1. Target Volume Concepts and Dose Metrics

The volumetric concepts in IGABT are largely defined by the GEC-ESTRO and ICRU 89 recommendations (Table 1) and provide a common language for reporting and comparing outcomes across centers and disease sites [10,13,14,31,32,33,34,35].

#### 3.1.1. Target Volume

For endometrial and vaginal cancers, these concepts are adapted to the disease site [12,17]. In postoperative endometrial cancer, the equivalent of the HR-CTV often includes the vaginal cuff and adjacent parametria at highest risk, whereas the IR-CTV may cover a lower vaginal segment or parametrial tissues depending on risk factors and the extent of prior external-beam radiotherapy [17,24,25,26]. In primary or recurrent vaginal cancer, the HR-CTV usually consists of the residual vaginal tumor with a small margin, while the IR-CTV may include the entire vaginal segment involved and surrounding paravaginal tissues [27,28,29] (Table 2).

#### 3.1.2. Imaging Modalities

**Figure 1:**
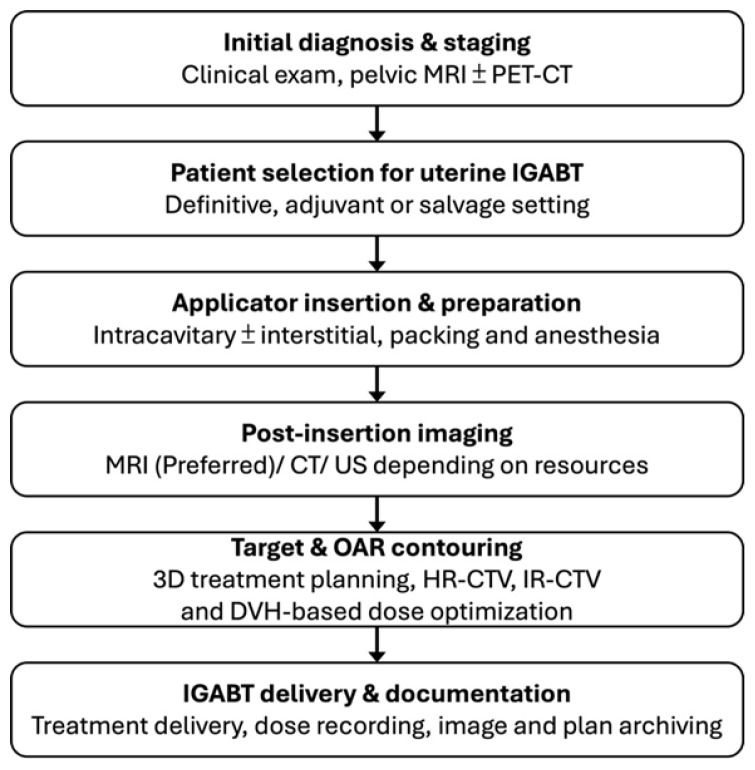
Workflow of uterine IGABT and imaging strategy.

**Figure 2:**
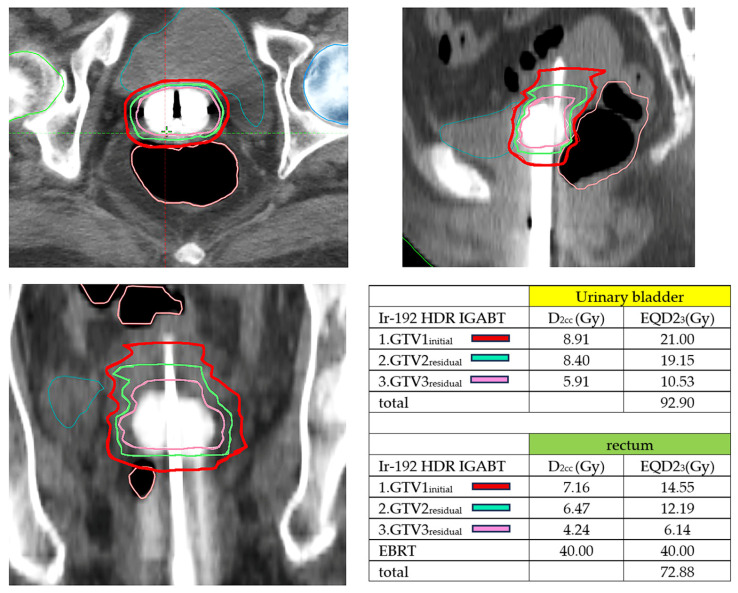
CT-based image-guided adaptive brachytherapy (IGABT) planning using a tandem-and-ovoid applicator across three high-dose-rate (HDR) Iridium-192 fractions for uterine cervix cancer. Axial (**upper left**), sagittal (**upper right**), and coronal (**lower left**) images demonstrate sequential target adaptive replanning. The initial gross tumor volume (GTV_1_, red) and residual volumes at the second (GTV_2_, green) and third (GTV_3_, pink) fractions illustrate progressive tumor regression. The corresponding D_2cc_ and EQD2_3_ values for each fraction and cumulative doses are summarized in the table (lower right panel), demonstrating a reduction in organ-at-risk dose for the bladder and rectum with adaptive treatment. D_2cc_: the dose delivered to the most exposed 2 cm^3^ of the organ-at-risk (OAR) wall; EQD2_3_ the equivalent dose in 2 Gy fractions (EQD2) calculated using an α/β ratio of 3 Gy; GTV1_initial: gross tumor volume at the first Iridium-192 IGABT fraction; GTV2_residual: residual gross tumor volume at the second IGABT fraction; GTV3_residual: residual gross tumor volume at the third IGABT fraction.

1. CT-based planning allows reliable visualization of applicator geometry and OARs but is limited by poorer soft-tissue contrast between tumor and normal tissues [6]. To mitigate this condition, contouring guidelines and atlases have been developed for CT-based IGABT, including MRI-informed surrogate landmarks for cervical and vaginal targets [7,19,33].
2. Hybrid imaging approaches help bridge the gap where routine MRI is not feasible for every fraction: [4,21,37]. CT + ultrasound (US): Transrectal or transvaginal ultrasound can guide applicator insertion, assess residual disease, and reduce the risk of uterine perforation, particularly in stenotic cervices or post-hysterectomy anatomy [39,40,41,42].
3. MRI-informed CT: A diagnostic or pre-brachytherapy MRI is used to define the initial extent and regression pattern of the tumor, which then serves as a guide for CT-based contouring at subsequent fractions [13,35,38,43,44,45]. This approach is applicable not only to cervical cancer but also to complex vaginal or paravaginal recurrences [19,27,28,29]. In routine practice, uterine IGABT follows a stepwise process that includes applicator insertion, post-insertion imaging, target and OAR contouring, and three-dimensional dose optimization. The overall workflow and imaging strategy are summarized in Figure 1. A CT-based Iridium-192 high-dose-rate (HDR) IGABT using a tandem and ovoid applicator for uterine cervical cancer is shown in Figure 2, demonstrating the corresponding D_2cc_ and EQD2_3_ values for each fraction; cumulative doses demonstrate a reduction in the organ-at-risk dose for the bladder and rectum with adaptive treatment.

### 3.2. Applicator Techniques

Applicator choice must be tailored to the disease site, extent, and geometry:

Intracavitary applicators (tandem-and-ring or tandem-and-ovoids applicators) are the backbone of cervical IGABT for centrally located tumors with limited parametrial extension [3,6,26]. In the postoperative setting, vaginal cylinders of appropriate diameter are commonly used for endometrial cancers with vaginal cuff involvement or as adjuvant treatment to the upper vagina [16,19].

Combined intracavitary/interstitial (IC/IS) applicators integrate a standard intracavitary component with interstitial needles or catheters inserted through the ring, ovoids, or perineal templates [3,40,46]. IC/IS techniques are particularly important for bulky, asymmetric, or deeply infiltrating disease (e.g., extensive parametrial involvement, lower vaginal extension, or paravaginal recurrence) where intracavitary applicators alone cannot adequately cover the HR-CTV without exceeding OAR dose constraints [6,19,36].

Purely interstitial implants using perineal templates may be required to select recurrent or de novo vaginal cancers, especially when prior surgery or radiation has significantly altered pelvic anatomy [3,19]. In these scenarios, MRI or high-quality CT guidance is critical for optimizing needle geometry relative to the tumor and OARs [4,21,33].

Across uterine malignancies, the integration of advanced imaging with flexible IC/IS and interstitial applicators allows clinicians to sculpt the dose to anatomically and biologically relevant targets [3,47]. Systematic documentation of applicator type, imaging modality, and resulting DVH metrics is essential to interpret reported outcomes and to refine IGABT strategies for cervical, endometrial, and vaginal cancers in a harmonized framework [19,48].

## 4. Discussion

### 4.1. IGABT for Locally Advanced Cervical Cancer

Locally advanced cervical cancer (LACC) has been the primary driver of the development and validation of image-guided adaptive brachytherapy [1,5,45]. The transition from conventional, radiograph-based intracavitary brachytherapy to volumetric, MRI- or CT-guided IGABT has reshaped standards of care by enabling individualized dose escalation to the residual tumor while accounting for organ-at-risk (OAR) constraints [5]. This paradigm has translated into a higher therapeutic ratio in terms of improved pelvic control, overall survival, and minimal toxicities compared with historical two-dimensional (2D) brachytherapy series and now provides a benchmark against which emerging techniques in other uterine malignancies are measured [16,36].

### 4.2. Historical Evolution from 2D-BT to 3D-IGABT

Historically, definitive chemoradiation for LACC was delivered using two-dimensional intracavitary brachytherapy, typically using tandem-and-ovoid or tandem-and-ring applicators with dose prescription for geometric reference points such as point A [7,8]. While this approach achieved reasonable local control, it lacked the ability to visualize the true extent of residual disease and OARs, leading to substantial inter-patient and inter-institutional variability in dose distributions and late toxicity [16].

The introduction of three-dimensional imaging into brachytherapy planning, initially with CT and subsequently with MRI, provided a means to move from point-based to volume-based prescription [6,7,8]. The GEC-ESTRO recommendations and the subsequent ICRU Report 89 formalized target volume concepts (GTV_res, HR-CTV, IR-CTV) and DVH-based reporting parameters such as D90 for targets and D_2cc_ for OARs, establishing a common language for planning and outcome analysis [6].

In parallel, multi-institutional initiatives such as the RetroEMBRACE study and the prospective EMBRACE trials were launched to evaluate chemoradiation combined with MRI- or CT-based IGABT in large, real-world cohorts of LACC patients [37,46]. RetroEMBRACE retrospectively analyzed 731 patients treated with image-guided brachytherapy (predominantly MRI-based) across 12 centers, while EMBRACE-I prospectively enrolled over 1400 patients treated with MRI-guided IGABT using standardized contouring and reporting [33].

Other institutional and national efforts, including the French STIC study and subsequent meta-analyses, compared 2D versus 3D brachytherapy and demonstrated improved local control and reduced severe morbidity with 3D image-based planning, even when controlling for concurrent chemotherapy and external-beam radiotherapy (EBRT) regimens [16]. These converging data, together with accumulating implementation reports from diverse practice settings, have led to a broad consensus that 3D image-guided brachytherapy should now be considered the standard of care for LACC [19].

### 4.3. Clinical Outcomes and Dose–Response Measures

The key clinical question in LACC has been whether volumetric IGABT can consistently deliver high local control across all stages, including bulky and advanced tumors, without prohibitive toxicity [16]. The RetroEMBRACE cohort provided early, multi-center evidence that chemoradiation combined with IGABT achieves excellent pelvic control and overall survival, with 3–5-year local control rates approaching or exceeding 90% overall and particularly favorable results in early- and intermediate-stage disease [16,35,40]. Analyses of patterns of failure showed a reduction in central pelvic relapses compared with historical 2D series, with residual failures occurring at an increasing rate outside the high-dose brachytherapy volume (e.g., nodal or distant sites) [6,15].

EMBRACE-I subsequently confirmed and extended these findings in a prospective setting. In more than 1400 patients treated with MRI-based IGABT, 5-year local control rates were in the low 90% range, with meaningful improvements even in FIGO stage III–IVA disease compared with older 2D-based reports [16]. Overall survival at 5 years was approximately three-quarters, despite the inclusion of advanced stages, and severe morbidity remained limited [16]. EMBRACE-II builds on this foundation with protocol-defined target doses and tighter OAR constraints, aiming to further refine the balance between tumor control and late toxicity [16,31].

A central strength of the IGABT paradigm is the ability to perform quantitative dose–response analyses [6,15]. RetroEMBRACE and related studies demonstrated that multiple series consistently demonstrate a strong association between HR-CTV D90 and the probability of durable local tumor control across tumor sizes: cumulative EQD2 doses ≥ ~85 Gy to the HR-CTV D90, delivered within an overall treatment time of about 7 weeks, are associated with 3-year local control rates above 90% for small and intermediate HR-CTV volumes, with somewhat lower but still favorable control in very large volumes [16,22]. These analyses also highlight HR-CTV volume at the time of brachytherapy as an independent prognostic factor, underscoring the importance of early and sustained tumor regression during EBRT and concurrent chemotherapy [1,16].

The evolution from purely intracavitary to combined intracavitary–interstitial (IC/IS) techniques has been crucial for maintaining dose–response advantages in larger or asymmetric tumors [3]. In RetroEMBRACE, centers that implemented IC/IS applicators were better able to deliver the desired HR-CTV D90 in patients with HR-CTV volumes ≥ ~30 cm^3^, with corresponding gains in local control and no significant increase in severe late morbidity [16]. These data underpin current recommendations that IC/IS IGABT should be strongly considered for bulky or parametrial extensive tumors, and they provide a template for similar dose-escalation strategies in other uterine malignancies [16,33].

### 4.4. Toxicity, Dose–Volume Constraints, and PRO/QoL

**Figure 3:**
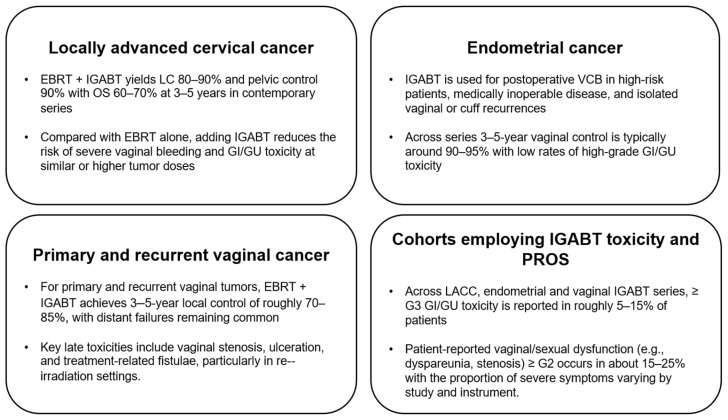
Summary of the recent literature on uterine IGABT outcomes and toxicity.

The transition to IGABT has not only improved tumor control but has also reshaped the profile of late morbidity [16,31]. An overview of key outcome and toxicity data across disease sites is provided in Figure 3.

In RetroEMBRACE and EMBRACE-I, the cumulative incidence of grade 3–5 late genitourinary, gastrointestinal, and vaginal toxicities was generally in the single-digit range per organ at 5 years, which is markedly lower than many historical 2D series [11,12,19,31]. The French STIC study and other comparative cohorts similarly reported that 3D image-based brachytherapy halves the rate of severe late toxicity compared with 2D planning, even when local control is improved [16,40].

These clinical gains are closely linked to dose–volume constraints derived from DVH analysis. ICRU 89 and professional society guidelines emphasize dose–volume parameters based on the most exposed 2 cm^3^ of critical organs—particularly the bladder, rectum, and sigmoid as the primary predictors of late severe toxicity—and recommend cumulative EQD2 thresholds that balance local control with organ preservation [16]. EMBRACE-based analyses have quantified dose–response relationships for specific endpoints such as late diarrhea, rectal bleeding, urinary morbidity, and vaginal stenosis, allowing clinicians to prioritize both target coverage and OAR protection during plan optimization [3,6,19,31].

Beyond physician-rated toxicity, prospective patient-reported outcome (PRO) data from EMBRACE have provided a nuanced view of long-term survivorship [15,41]. Longitudinal analyses using EORTC QLQ-C30 and CX24 questionnaires show that global health status and many functional domains remain stable or recover over time in most patients, supporting the notion that IGABT-based chemoradiation is compatible with acceptable overall quality of life [4,20,46]. However, subsets of patients experience persistent moderate-to-severe symptoms, particularly in bowel, bladder, and sexual domains, and these persistent symptoms are strongly associated with impaired quality of life and workability [4].

Importantly, PRO analyses have begun to link specific DVH parameters and clinical risk factors to patient-reported symptom trajectories, suggesting that further refinement of dose constraints and better management of comorbidities, treatment breaks, and supportive care may reduce the burden of late morbidity [6,16,31,46,47]. In this context, IGABT offers not only a tool for improving local control but also a platform for personalized toxicity mitigation—an approach that will be increasingly relevant as similar concepts are extended to endometrial and vaginal cancers [16,49,50,51,52].

### 4.5. IGABT in Endometrial and Vaginal Cancers

Compared with locally advanced cervical cancer, the evidence base for image-guided brachytherapy in endometrial and vaginal cancers is smaller and more heterogeneous, but it is expanding rapidly [19]. Most available data come from retrospective single- or multi-institutional series that integrate three-dimensional (3D) planning—often CT-based and increasingly MRI-assisted—with high-dose-rate (HDR) intracavitary or interstitial techniques. Overall, these studies suggest that applying IGABT principles beyond the cervix can provide excellent local control with acceptable toxicity in both postoperative and definitive settings for endometrial cancer and in primary or recurrent vaginal cancer [17,24,25,26,27,28,29].

### 4.6. Postoperative Adjuvant Brachytherapy for Endometrial Cancer

Adjuvant vaginal cuff brachytherapy (VCB) is a standard component of postoperative radiotherapy for many patients with early-stage, high-intermediate-risk endometrial cancer [16,19]. The PORTEC-2 trial established VCB alone as non-inferior to pelvic external-beam radiotherapy (EBRT) for vaginal control with lower rates of gastrointestinal toxicity, thereby cementing VCB as the preferred option for selected patients [16,19,53]. Historically delivered using 2D planning and single-channel cylinders, VCB has increasingly adopted 3D image-based planning, with CT or MRI used to confirm applicator position, vaginal cuff coverage, and organ-at-risk (OAR) doses [6,19].

In 3D IG-VCB, the clinical target volume typically includes the upper 3–5 cm of the vagina and adjacent parametria, with individualization based on surgical and pathologic risk factors [16]. Single-channel or multi-channel cylinders are selected to optimize contact with the vaginal mucosa and to allow for dose shaping in cases of asymmetric risk [19]. Typical HDR schedules deliver an equivalent dose in 2 Gy fractions (EQD2) of approximately 20–30 Gy to the high-risk vaginal cuff region, in addition to prior EBRT when used, while accounting for OAR constraints for the bladder, rectum, and sigmoid [19].

Several series have highlighted the value of CT- or MRI-informed planning for VCB [12,13,14,15,47]. MRI-based evaluations of VCB have identified substantial under-coverage of the vaginal cuff when relying on CT or standard planning assumptions alone, suggesting that image guidance may help reduce marginal underdosage, particularly in patients with complex post-surgical anatomy or narrow vaginas [6,19]. Early reports of image-guided VCB in high-intermediate and high-risk cohorts show very low isolated vaginal recurrence rates and favorable toxicity profiles, though follow-up remains relatively limited and prospective PRO data are scarce [19,38,40,51].

### 4.7. IGABT for Primary and Recurrent Vaginal Cancers

Primary vaginal cancer is rare, accounting for a small fraction of gynecologic malignancies, but brachytherapy is a cornerstone of its curative management [19,36]. Tumor shrinkage during EBRT, combined with the confined anatomy of the vagina, makes this disease particularly well suited to image-guided adaptive brachytherapy [37].

GEC-ESTRO ACROP recommendations define target concepts for primary vaginal cancer IGABT, adapting GTV_res, HR-CTV, and IR-CTV to vaginal anatomy while emphasizing the need to cover the whole vaginal segment involved and at-risk paravaginal tissues [19,33]. Clinically, a variety of applicator configurations are used—ranging from multi-channel vaginal cylinders to perineal template-based interstitial implants—depending on tumor length, thickness, and circumferential involvement [3,19]. CT- or MRI-assisted planning allows dose sculpting to mitigate eccentric or bulky disease while accounting for bladder, rectal, and urethral constraints [6,54].

Multi-center and single-institution series of image-guided brachytherapy for primary or recurrent vaginal cancer have reported 3–5-year local control rates in the range of approximately 70–90%, with better outcomes for early-stage, limited-length lesions and worse outcomes for extensive or proximal tumors [16]. Late grade 3 or higher GI/GU/vaginal toxicity rates are generally acceptable and appear lower than in historical 2D cohorts, particularly when modern fractionation and OAR D_2cc_ constraints are applied [19]. Recent MRI-guided HDR brachytherapy series further support high rates of local control with limited high-grade toxicity, while highlighting that distant failure remains a major cause of treatment failure, reinforcing the need for optimized systemic therapy and multidisciplinary care [16].

Evidence specific to patient-reported outcomes and quality of life in vaginal cancer is limited [4,19]. Available data suggest that sexual dysfunction, vaginal stenosis, and dyspareunia can be clinically significant, particularly in patients requiring high total doses or extensive interstitial implants [3,19]. These findings mirror experience from cervical IGABT and underscore the importance of structured survivorship programs, including vaginal dilator use, lubricants, hormonal therapy when appropriate, and psycho-sexual counseling [19].

### 4.8. Comparative Outcomes and Evidence Gaps

Taken together, early IGABT experiences in endometrial and vaginal cancers indicate that the core principles established for LACC—volumetric target definition, DVH-based dose prescription, and careful OAR constraint management—can be successfully translated to other uterine malignancies [7,19]. Postoperative image-guided VCB provides excellent vaginal control with low toxicity in appropriately selected endometrial cancer patients; definitive IGABT for medically inoperable endometrial cancer achieves high uterine and locoregional control with acceptable morbidity; and IGABT for primary or recurrent vaginal cancer yields local control rates that compare favorably with historical 2D series [16].

Nonetheless, substantial evidence gaps remain. Most studies are retrospective, involve relatively small patient cohorts, and span long time periods with evolving imaging, applicator, and systemic therapy techniques [36]. There is limited information on long-term PROs and quality of life, particularly regarding sexual function and body image in endometrial and vaginal cancer survivors [4,19]. Prospective, disease-specific registries and trials integrating standardized target concepts, DVH reporting, and PRO instruments are needed to refine dose–response relationships, optimize dose–fractionation schedules for different risk groups, and better define the role of IC/IS IGABT in complex presentations and re-irradiation settings [22].

In this context, applying a unified IGABT framework across cervical, endometrial, and vaginal malignancies offers a unique opportunity: experience and dose–response data from the largest cohort—LACC—can inform rational dose escalation and constraint setting in rarer disease sites, while emerging data from endometrial and vaginal cancers can, in turn, refine our understanding of organ tolerance and survivorship outcomes across the uterine cancer spectrum [19].

### 4.9. Technical Challenges and Emerging Directions

Despite the substantial advances achieved with image-guided adaptive brachytherapy, several technical and organizational challenges still limit its broad adoption and refinement across uterine malignancies [55]. These challenges span workflow and logistics, dose accumulation and optimization strategies, fractionation and overall treatment time (OTT), and the integration of functional imaging, biological information, and artificial intelligence (AI) into routine practice [6,22,47]. Addressing these issues will be critical for further improving outcomes and disseminating IGABT beyond highly specialized centers [46].

### 4.10. Workflow, Logistics, and Dose Accumulation

MRI- and CT-based IGABT utilizes a complex, resource-intensive workflow that involves close coordination between radiation oncologists, anesthesiologists, radiologists, physicists, and specialized nursing staff. Detailed implementation reports from high-volume centers describe multi-step processes that include applicator insertion (often under anesthesia), image acquisition, target and organ-at-risk (OAR) delineation, treatment planning, quality assurance, and delivery—typically within a constrained time window on the day of brachytherapy [4,51,52,53]. These experiences demonstrate that MRI-based interstitial and intracavitary–interstitial workflows are feasible and can be streamlined, but they also highlight the need for robust institutional commitment and training [3].

Several groups have proposed practical guidance on how to implement MRI-based IGABT, including recommendations on imaging protocols, applicator reconstruction, contouring standardization, and physics preparation [33]. Hybrid workflows—such as MRI for the first fraction followed by CT-based planning for subsequent fractions, or CT combined with ultrasound guidance—have been introduced to reduce MRI demand while maintaining acceptable target definition and sparing OARs [4,6,21,37]. Experience from national and regional initiatives shows that stepwise implementation, beginning with CT-based 3D brachytherapy and gradually integrating MRI and interstitial techniques, can effectively transition centers and even entire health systems to advanced image-guided adaptive approaches [3,12,20,27].

A related technical challenge is accurate dose accumulation across external-beam radiotherapy (EBRT) and multiple brachytherapy fractions. In most clinical series, cumulative doses to targets and OARs are estimated by simple DVH parameter addition in EQD2, assuming rigid anatomy and consistent organ filling [53]. This approach is straightforward and has underpinned the development of dose–response models and D_2cc_ constraints in IGABT [16,55,56,57,58]. However, it does not fully account for interfraction anatomical changes or the complex deformations induced by brachytherapy applicators.

Deformable image registration (DIR) has been proposed to enable “4D brachytherapy,” in which voxel-wise doses from EBRT and each brachytherapy fraction are mapped to a common reference anatomy for more accurate dose accumulation and biological modeling [16,55,57,58]. Early studies have demonstrated the feasibility of DIR-based dose summation and have explored its impact on OAR DVH parameters; however, they also reveal uncertainties in registration accuracy, particularly in regions of steep dose gradients and substantial deformation [6,12,33,56]. At present, DIR remains primarily a research tool; most guidelines still recommend simple DVH parameter addition for routine reporting, while encouraging further work to standardize and validate 4D brachytherapy approaches [7,20].

### 4.11. Fractionation Strategies and Overall Treatment Time

High-dose-rate (HDR) IGABT for cervical and other uterine cancers is delivered using a variety of fractionation schedules, reflecting historical practice, institutional preferences, and differing philosophies about balancing the target dose, sparing OARs, and mitigating logistical constraints [22,31]. Common regimens combine EBRT (with or without nodal boosts) and HDR brachytherapy in 3–5 fractions, with per-fraction doses tailored to achieve a cumulative HR-CTV D90 in the desired EQD2 range while accounting for D_2cc_ constraints [6].

Across multiple series and prospective cohorts, overall treatment time is an independent predictor of local control and survival in locally advanced cervical cancer, with most guidelines recommending completion of EBRT and brachytherapy within approximately 7–8 weeks [16]. Prolonged OTT is associated with inferior pelvic control, likely reflecting accelerated repopulation and the cumulative impact of interruptions to treatment [16,41,58]. In this context, fractionation choices and workflow efficiency are tightly coupled: schedules that require multiple implants or separate imaging sessions must be balanced against the risk of delays, particularly in resource-limited settings [22].

The EMBRACE-II protocol has sought to harmonize fractionation and dose objectives by defining target EQD2 ranges for HR-CTV and IR-CTV, as well as upper limits for OAR D_2cc_ values, within a standardized chemoradiation backbone and recommended OTT [22]. Similarly, though less formalized, principles are increasingly applied to endometrial and vaginal IGABT, where cumulative doses are adapted to surgical status, prior irradiation, histology, and patterns of spread [19,30,31]. Nevertheless, high-quality comparative data for different brachytherapy fractionation schedules—particularly in non-cervical uterine malignancies—remain limited [22]. Prospective registries and collaborative planning studies will be necessary to refine fractionation strategies across risk groups and to optimize the trade-off between local control, late morbidity, and operational feasibility [15,16].

### 4.12. Functional Imaging, Biological IGABT, AI, and Radiomics

Beyond anatomical imaging, functional MRI techniques such as diffusion-weighted imaging (DWI), dynamic contrast-enhanced MRI (DCE-MRI), and quantitative T2 mapping offer insight into tumor cellularity, perfusion, and microenvironmental characteristics [20]. Multiple single-center studies have reported associations between pre-treatment or early intra-treatment DCE-MRI parameters and local control or disease-free survival in locally advanced cervical cancer, suggesting that poorly perfused or hypoxic tumors may be at higher risk of failure [7,16,53,58].

The ongoing IQ-EMBRACE study systematically incorporates quantitative MRI into the IGABT workflow across multiple centers, acquiring standardized DWI, DCE, and T2 mapping sequences and conducting centralized analysis [5,6,7,16,33]. Its goals are to identify robust imaging biomarkers that predict response and toxicity, evaluate the reproducibility of quantitative metrics, and lay the groundwork for “biological IGABT” in which dose prescription or target delineation may be adapted according to functional imaging signatures [7,21,30]. Parallel initiatives such as BIOEMBRACE explore integration of imaging with circulating and tissue biomarkers, further broadening the potential for biologically guided personalization [44,45].

Radiomics and machine-learning approaches applied to pre-treatment and pre-brachytherapy MRI are also emerging. Recent analyses using first-order and radiomic texture features from T2-weighted and diffusion-weighted images have identified patterns associated with local control, progression-free survival, and treatment response in locally advanced cervical cancer, although most studies are small and retrospective [16]. At present, radiomics signatures remain exploratory; they require rigorous external validation, harmonization of imaging acquisition, and careful assessment of clinical utility before being integrated into routine IGABT workflows or prospective dose-escalation strategies [56].

In parallel, artificial intelligence is rapidly being explored to support IGABT planning [6]. Deep learning-based auto-segmentation models have been developed to delineate HR-CTV and pelvic OARs on CT and MRI for cervical brachytherapy, using convolutional neural networks and U-Net-like architectures [4,21,33]. Early evaluations show that AI-generated contours can substantially reduce contouring time and inter-observer variability; however, clinically acceptable plans still require careful review and manual adjustment, especially in complex post-surgical or recurrent anatomy [33]. AI-driven tools are also being investigated for applicator reconstruction, dose prediction, and semi-automatic plan optimization in HDR brachytherapy [6].

From a broader radiotherapy perspective, professional societies and cooperative groups have emphasized that AI tools must be introduced within robust quality assurance frameworks, including systematic validation, version control, and monitoring of performance over time and across populations [4]. Heterogeneity in imaging protocols, applicator types, and patient anatomy across centers poses particular challenges for generalizing AI models developed in single institutions [2]. Additionally, regulatory considerations and concerns about algorithmic bias and transparency must be addressed before AI can be relied upon for high-stakes decisions in IGABT.

For endometrial and vaginal cancers, functional imaging, radiomics, and AI applications are even less mature than in cervical cancer, largely due to lower incidence and limited datasets [19]. Nonetheless, the same conceptual framework applies where functional MRI and radiomic signatures could eventually help identify patients at high risk of local or distant failure, while AI-based segmentation and planning tools may be especially valuable in complex recurrent cases or re-irradiation [5,6,16,31]. Multi-institutional collaboration and integration with existing cervical IGABT research platforms will be essential to accelerate progress in these rarer uterine malignancies [47].

## 5. Conclusions

Image-guided adaptive brachytherapy has redefined the standard of care for the management of locally advanced cervical cancer. Through the integration of volumetric target concepts, DVH-based dose reporting, and advanced imaging, IGABT has enabled consistent dose escalation to the residual tumor while accounting for organ-at-risk constraints, resulting in high local control rates and reduced severe morbidity compared with historical 2D brachytherapy. These advances provide a robust clinical and technical foundation for the broader application of IGABT principles across uterine malignancies.

Emerging data in endometrial and vaginal cancers indicate that the same framework—careful definition of HR-CTV and IR-CTV, cumulative EQD2-based prescription, and systematic OAR constraint management—can be successfully adapted to postoperative vaginal cuff brachytherapy, definitive treatment of medically inoperable endometrial cancer, and primary or recurrent vaginal tumors. Although the evidence base is smaller and more heterogeneous than in cervical cancer, contemporary 3D IGABT series report excellent local control and acceptable toxicity in both adjuvant and definitive settings, suggesting substantial potential to improve outcomes in less common uterine malignancies.

At the same time, long-term considerations for survivorship are increasingly recognized as central to treatment evaluation. Prospective cohorts and narrative syntheses suggest that IGABT-based chemoradiation is compatible with generally favorable global quality of life for many patients, but that a subset experiences persistent gastrointestinal, genitourinary, and sexual symptoms that significantly affect daily functioning. Linking detailed DVH parameters, clinical risk factors, and patient-reported outcomes will be crucial for refining dose–volume constraints and developing strategies to prevent and manage late effects in cervical, endometrial, and vaginal cancer survivors.

Looking ahead, functional and quantitative imaging, radiomics, and AI offer promising avenues for further personalization of IGABT. Quantitative MRI techniques may help identify biologically unfavorable tumors and inform adaptive dose escalation or target modification, while radiomic signatures and machine-learning models could support risk stratification and prediction of toxicity. AI-assisted auto-segmentation and semi-automatic planning tools have the potential to reduce contouring time and inter-observer variability, although rigorous external validation and QA frameworks are essential before they can be fully integrated into routine practice.

## Figures and Tables

**Table 1:** Target volume definition for IGABT.

Target Volume	Definition
Gross Tumor Volume (GTV):	Forms the basis for treatment prescription and planning. Clinical, imaging, and/or pathology investigations provide information on the macroscopic extent of disease for the primary tumor [10]. It is the most important prognostic factor for survival and local control [14]
Gross Tumor Volume (GTV_res)	The residual macroscopic or microscopic tumor volume and topographical change at the time of brachytherapy after treatment, integrating clinical examination and imaging [5,8,10,14].
High-Risk Clinical Target Volume (HR-CTV):	Encompasses the GTV_res and the entire cervix, plus contiguous regions at high risk of harboring residual microscopic disease (e.g., parametrial or vaginal extension that has regressed but remains at risk) [12]. The intent of the HR-CTV is to deliver the highest possible dose (≥84 Gy) to eradicate all residual disease [7,10,14].
Intermediate-Risk Clinical Target Volume (IR-CTV):	Encompasses the GTV_res and the entire cervix, plus contiguous regions at high risk of harboring residual microscopic disease (e.g., parametrial or vaginal extension that has regressed but remains at risk) [12]. The intent of the HR-CTV is to deliver the highest possible dose (at least ≥84 Gy) to eradicate all residual disease [7,10,14].

**Table 2:** Target volume definitions and dose–volume objectives in uterine IGABT.

Disease Site/Setting	Main Target Volumes and Key Definition Points	Typical Imaging and Applicators	Example Prescription and Total HR-CTV D90 (EQD2, α/β = 10)	Typical OARs to Report and Example D_2cc_ Constraints (EQD2, α/β = 3)	Key Guidelines/Protocols
Locally advanced cervical cancer (definitive)	GTV_res: residual gross tumor at BT; HR-CTV: entire cervix + residual disease + involved parametria; IR-CTV: HR-CTV + regions at risk (e.g., medial parametria, upper vagina).	MRI-based IGABT preferred; CT- or hybrid MRI/CT-based planning acceptable; tandem–ring/tandem–ovoid ± interstitial needles.	EBRT ~45–50.4 Gy + BT to reach HR-CTV D90 ≈ 85–90 Gy EQD2.	Bladder D_2cc_ ≤ ~90 Gy; rectum and sigmoid D_2cc_ ≤ ~70–75 Gy; bowel D_2cc_ ≤ ~65–70 Gy.	GEC-ESTRO, ICRU 89, EMBRACE protocols [10,23,36,37,38]
Endometrial cancer—postoperative vaginal cuff	HR-CTV: upper 3–5 cm vaginal cuff ± parametrial/paravaginal tissues depending on risk; IR-CTV: HR-CTV + margin along vagina.	CT- or MRI-based planning; single- or multi-channel vaginal cylinder, ovoids, or IC/IS vaginal applicator.	EBRT 45–50.4 Gy (if given) + BT to reach HR-CTV D90 ≈ 60–70 Gy EQD2 (risk-adapted).	Bladder and rectum D_2cc_ typically kept ≤ ~65–70 Gy; sigmoid/bowel ≤ ~60–65 Gy; vaginal wall points or D0.1cc recorded.	ABS and GEC-ESTRO EC recommendations [17,24,25,26]
Endometrial cancer—medically inoperable/definitive	HR-CTV: entire uterine cavity ± cervical involvement; IR-CTV: uterus + parametrial/vaginal at-risk regions.	MRI- or CT-based planning; tandem–ring/tandem–ovoid ± interstitial needles; image-guided uterine applicators.	EBRT 45–50.4 Gy ± boost + BT to HR-CTV D90 ≈ 70–80 Gy EQD2 depending on stage/volume.	Similar GI/GU constraints identified as LACC; urethra and vaginal wall doses are recorded when vaginal involvement is present.	ABS, institutional IGABT series [17,24,25,26]
Primary vaginal cancer	HR-CTV: gross vaginal tumor + involved wall/parametria; IR-CTV: involved segment + proximal/distal margins along vagina.	MRI- or CT-based planning; multi-channel cylinder or IC/IS vaginal template/applicator.	EBRT 45–50.4 Gy + BT to HR-CTV D90 ≈ 70–80 Gy EQD2 (site- and length-adapted).	Bladder/rectum/sigmoid/bowel D_2cc_ similar to cervical; urethral and vaginal wall doses explicitly reported.	GEC-ESTRO vaginal IGABT concepts, key series [26,27,28]
Recurrent vaginal/vaginal cuff disease	HR-CTV: recurrent lesion in cuff or vagina; IR-CTV: recurrent site + surgical bed/scar and margins.	MRI- or CT-based planning; multi-channel cylinder or IC/IS template; may require tailored interstitial implantation.	EBRT re-irradiation individualized; BT HR-CTV D90 often ≈ 60–75 Gy EQD2 depending on prior dose and OAR tolerance.	OAR constraints are individualized based on prior RT; cumulative bladder/rectum/sigmoid/bowel D_2cc_ reported (EQD2).	Salvage IGABT series, re-irradiation guidelines [26,27,28]

## Data Availability

No new data were created, all the data used for this review are publicly available in PUBMED or referencing the DOI number.

## Abbreviations

IGABT	image-guided brachytherapy
LACC	locally advanced cervical cancer
DVH	dose–volume histogram
MRI	magnetic resonance imaging
CT	computed tomography
BT	brachytherapy
ICRU	International Commission on Radiation Units and Measurements
MeSH	medical subject headings
GTV_res	gross tumor volume residual
HR-CTV	high-risk clinical target volume
IR-CTV	intermediate-risk clinical target volume
EQD2	equivalent dose in 2 Gy fractions
D_2cc_	dose to the most exposed 2 cm^3^
HR-CTV D90	minimum dose received by 90% of the HR-CTV
OAR	organ at risk
IC/IS	intracavitary/interstitial
EBRT	external-beam radiotherapy
PRO	patient-reported outcome
QoL	quality of life
HDR	high-dose rate
VCB	vaginal cuff brachytherapy
GEC-ESTRO ACROP	Groupe Européen de Curiethérapie of European Society for Radiotherapy and Oncology Advisory Committee for Radiation Oncology Practice
GI/GU	gastrointestinal/genitourinary
OTT	overall treatment time
DIR	deformable image registration
DWI	diffusion-weighted imaging
DCE-MRI	dynamic contrast-enhanced MRI
